# Influence of the Inducer on the Performance of a Miniature High-Speed Centrifugal Pump

**DOI:** 10.3390/mi16080952

**Published:** 2025-08-19

**Authors:** Yifu Hou, Xiaonian Zeng, Yuchuan Wang

**Affiliations:** College of Water Resources and Architectural Engineering, Northwest A&F University, Yangling 712100, China; zengxn@nwafu.edu.cn

**Keywords:** miniature high-speed centrifugal pump, inducer, cavitating flow, hydraulic performance

## Abstract

The inclusion of an inducer is an effective approach to improve the cavitation performance of centrifugal pumps, significantly influencing both the internal flow characteristics and the external performance of the pumps. This study examines a miniature high-speed centrifugal pump (MHCP) using numerical simulations based on the *k*-*ε* turbulence model, comparing the cases with an inducer and without one. Experimental tests on the pump’s external performance are conducted and flow visualization images are presented to validate the findings. The effects of the inducer on the tip leakage backflow, cavitation performance, and external pump performance are analyzed. The results show that the inducer provides pre-pressurization of the fluid, leading to a higher circumferential velocity at the impeller inlet and a reduced inlet flow angle. This allows for a reduction in the impeller blade inlet angle, resulting in smoother flow streamlines inside the impeller. Moreover, the inducer helps to suppress local low-pressure regions caused by the vortex and cavities generated by the interaction between the tip clearance backflow and the main flow, thereby mitigating cavitation in the non-blade zone. Within the investigated operating range, the pump with an inducer exhibits a significantly improved external hydraulic performance, including an increased head and efficiency, a reduced required net positive suction head (NPSHr), and a broader stable operating range.

## 1. Introduction

With the rapid advancement of unmanned aerial vehicle (UAV) technologies, there is a growing demand for increased payload capacities and enhanced electronic control systems [1,2]. However, improvements in UAV flight performance have led to significantly higher thermal loads generated by the complex onboard electronics, batteries, and motors [3]. To mitigate the adverse effects of thermal load on UAV functionality, reliable cooling solutions are required [4]. Liquid cooling systems, originally developed for data center thermal management, have recently been adapted for UAV applications, where centrifugal pumps are employed as the primary driving components for coolant circulation [5]. The performance of the centrifugal pump plays a pivotal role in determining the overall efficiency and stability of a thermal management system.

Numerous studies have focused on the internal flow characteristics of centrifugal pumps. Baun et al. conducted a study on the effect of the impeller–volute relative position on the hydraulic performance and radial hydraulic forces in a centrifugal pump. They found that optimizing this relative position can significantly enhance pump efficiency [6]. Shi et al. carried out numerical simulations to explore the pressure fluctuation mechanisms resulting from the interaction between the impeller and the diffuser [7]. The radial force characteristics of a single-volute vaneless centrifugal pump were examined by González et al. through a combination of numerical simulations and experimental tests [8]. By analyzing the internal pressure distribution and unsteady pressure fluctuations, they aimed to accurately predict the radial force acting on the pump. Barrio et al. further explored the unsteady flow caused by the impeller–volute interaction and successfully predicted the dynamic radial forces and torque at the blade-passing frequency [9]. In addition, Fernández et al. conducted a deterministic decomposition of the unsteady flow in a centrifugal pump, uncovering how the radial clearance between the impeller outlet and the volute tongue affects the flow structure between the impeller blades [10].

Cavitation is a common issue in centrifugal pumps, particularly under high rotating speeds and high flow rate conditions [11]. As the inlet velocity increases, the local pressure at the impeller inlet may drop below the vapor pressure of the fluid, initiating cavities formation. These cavities reduce the effective flow area within the passage and, under severe conditions, may completely obstruct fluid flow, leading to pump performance degradation or even mechanical failure [12]. To address these challenges, extensive numerical and experimental investigations have been conducted to better understand the cavitation characteristics of centrifugal pumps [13]. Wang et al. analyzed the characteristics of irreversible energy losses induced by cavitating flows in centrifugal pumps based on entropy generation theory and detailed flow field investigations. Their study revealed that turbulent velocity fluctuations are the primary contributors to increased total entropy generation, and that high-speed vortex structures have a significant impact on irreversible flow losses [14]. The pressure pulsations arising from quasi-steady cavitation in centrifugal pumps were analyzed by Lu et al. through combined numerical and experimental methods. Their work identified the onset of cavitation as well as the critical point at which cavitation becomes unstable [15]. The variations in flow losses and internal flow characteristics of low specific speed centrifugal pumps under different cavitation scenarios were analyzed by Jia et al. Additionally, they experimentally examined the corresponding changes in the hydraulic characteristics of the pumps [16]. Yan et al. performed comparative numerical simulations to investigate the effects of annular and straight inlet configurations on the cavitation characteristics of inducers [17,18]. Parikh et al. conducted combined numerical and experimental investigations to evaluate the impact of inducer integration on centrifugal pump performance, and their results confirmed that the inducer effectively enhances the cavitation resistance of centrifugal pumps [19,20]. High-speed imaging was employed by Mansour et al. to study the effects of an inducer on centrifugal pumps equipped with semi-open impellers [21]. Furthermore, Samanody et al., based on experimental studies, demonstrated that for high-speed centrifugal pumps, helical inducers outperform axial inducers in terms of overall performance [22]. The internal flow stability in an MHCP was analyzed by Zhou et al. with respect to variations in the tip clearance. Using flow visualization techniques, they successfully observed cavitation vortices forming in the tip clearance region of inducer [23]. Building upon the experimental platform established by Zhou at Xi’an Jiaotong University, this research conducted a more comprehensive investigation into the hydraulic and cavitation performance of an MHCP.

Based on the literature review, it is evident that the existing studies on cavitation in centrifugal pumps have primarily focused on configurations without an inducer. Investigations into the effects of inducers on the cavitation performance of MHCPs remain limited. A numerical model of an MHCP was established in this work and verified against experimental measurements. The study explored how an inducer alters the distribution of major physical parameters inside the flow field and examined its role in shaping the cavitation behavior of the pump. In addition, performance experiments were carried out to assess the inducer’s effect on the pump’s hydraulic efficiency and head characteristics.

## 2. Simulation Approach and Experimental Validation

### 2.1. Governing Equations

The governing equations form the foundation for numerical simulations of MHCPs, describing both the fluid dynamics and thermodynamic behavior. By resolving the continuity, momentum, and energy equations, the flow field can be effectively analyzed, including the variations in and the spatial distributions of velocity, pressure, and temperature. For incompressible fluids, where the density is constant, the fundamental governing equations in fluid dynamics are the continuity equation and the Navier–Stokes momentum equation [24,25].

The energy conservation equation can be neglected in this study, as the flow within the centrifugal pump is considered incompressible and the temperature variation is negligible. Therefore, thermal effects have a minimal impact on the flow behavior and are not accounted for in the numerical simulation.

### 2.2. Turbulent Model

The *k*-*ε* turbulence model is one of the most widely used two-equation models for engineering applications. For incompressible fluids, the standard form of the turbulence transport equations is given by

(1) $$\frac{\partial }{\partial t}(\rho k)+\frac{\partial }{\partial x_{j}}\left[\rho u_{j}k-\left(\mu +\frac{\mu_{t}}{\sigma_{k}}\right)\frac{\partial k}{\partial x_{j}}\right]=\rho \left(p_{k}-\varepsilon \right)$$

(2) $$\frac{\partial }{\partial t}(\rho \varepsilon )+\frac{\partial }{\partial x_{j}}\left[\rho u_{j}\varepsilon -\left(\mu +\frac{\mu_{t}}{\sigma_{k}}\right)\frac{\partial k}{\partial x_{j}}\right]=\rho \frac{\varepsilon }{k}\left(C_{1}p_{k}-C_{2}\varepsilon \right)$$

### 2.3. Cavitation Model

The Zwart–Gerber–Belamri cavitation model is adopted in this study to simulate the cavitation phenomena in the centrifugal pump. The model characterizes the vaporization and condensation processes through the mass transfer source terms.

When the local pressure *p* is less than or equal to the saturation vapor pressure *p*_*v*_, vapor bubbles are generated from the liquid, and the evaporation source term is defined as follows:

(3) $${\dot{m}}^{-}=F_{vap}\frac{3\alpha_{ruc}\rho_{v}\left(1-\alpha_{v}\right)}{R_{B}}\sqrt{\frac{2\left(p_{v}-p\right)}{3\rho_{1}}}$$

When the local pressure *p* > *p*_*v*_, vapor bubbles condense into a liquid, and the condensation source term is expressed as follows:

(4) $${\dot{m}}^{+}=F_{\mathrm{cond}}\frac{3\alpha_{v}\rho_{v}}{R_{B}}\sqrt{\frac{2\left(p-p_{v}\right)}{3\rho_{1}}}$$

In the equations, ${\dot{m}}^{-}$ represents the mass transfer rate during bubble generation (vaporization); ${\dot{m}}^{+}$ indicates the mass transfer rate during bubble collapse (condensation); $F_{vap}$ is the empirical coefficient for the vaporization source term; $F_{\mathrm{cond}}$ is the empirical coefficient for the condensation source term; $\alpha_{ruc}$ denotes the nucleation site volume fraction; $\alpha_{v}$ is the vapor volume fraction; and $R_{B}$ is the average bubble diameter, set to 2 × 10^−6^ m in this study.

### 2.4. Computation Domain and Setup

**Figure 1:**
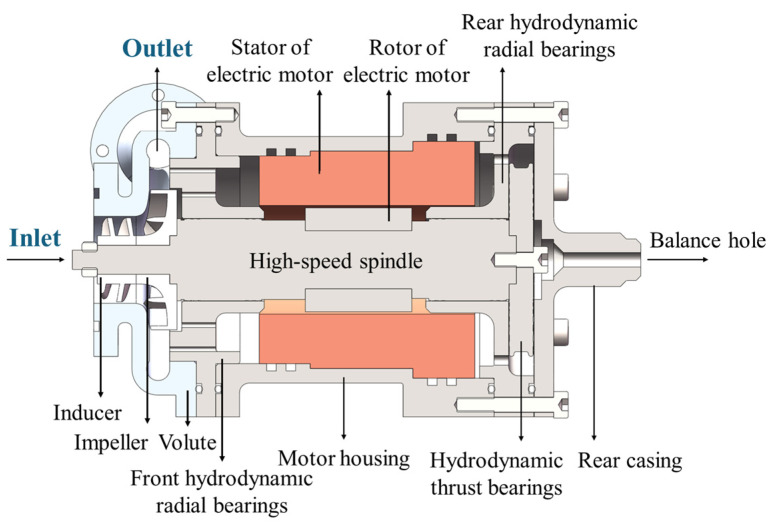
Cross-sectional structure of the MHCP.

**Figure 2:**
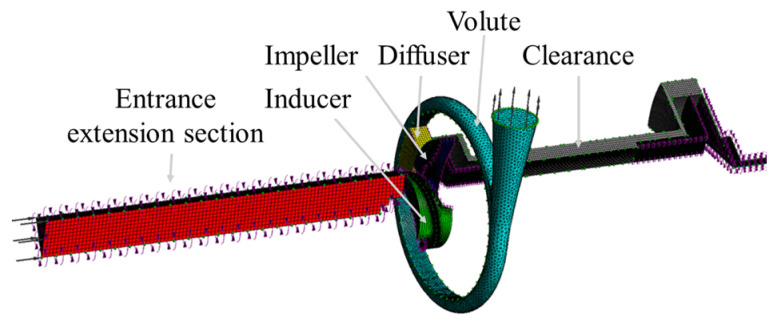
Numerical computational domain of the MHCP.

A centrifugal pump was selected as the subject of this study. It was designed with a flow rate of *Q* = 0.98 m^3^/h, a head of *H* = 30 m, and a rotational speed of *n* = 19,000 rpm, corresponding to a specific speed of *n*_s_ = 76. The structural cross-section of the pump is illustrated in Figure 1, and the internal fluid domain is shown in Figure 2. The computational domain mainly includes the inlet section, inducer, impeller, clearance flow passage, and volute. The detailed geometric parameters of the pump can be found in our previously published work [23].

In the numerical simulation, a pressure inlet and a mass flow outlet were applied as the boundary conditions. All the walls within the computational domain were treated as no-slip boundaries, and the interface between the rotating and stationary components was handled using the Frozen Rotor approach. The convergence criterion was set to a residual level of 10^−5^. Additionally, the pressure difference between the inlet and outlet, as well as the shaft power, were monitored during the computation. The simulation was considered converged once these monitored values stabilized without noticeable fluctuations.

**Figure 3:**
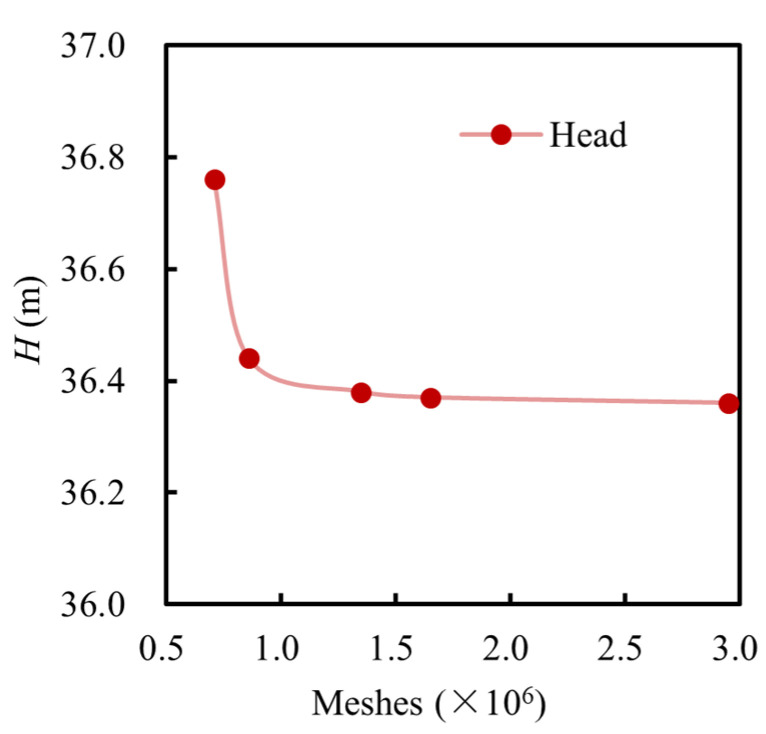
Grid independence verification.

The fluid domain was meshed using ICEM CFD 2022 and ANSYS TurboGrid 2022. To ensure mesh quality, additional refinement was applied in the critical regions, such as the blade surface, tip clearance of 0.3 mm, and near-wall areas. The near-wall mesh was refined to maintain *y*^+^ values below 80, thereby ensuring an accurate resolution of the boundary layer flow. Moreover, to assess the grid independence, five different mesh densities were generated for the steady-state simulations. The total number of mesh elements was set to 0.73 million, 0.94 million, 1.38 million, 1.70 million, and 2.95 million, yielding corresponding head values of 36.77 m, 36.44 m, 36.40 m, 36.39 m, and 36.38 m, respectively, as depicted in Figure 3. As the mesh density increased, the predicted head declined and then leveled off, demonstrating that the numerical solution has converged. Considering the balance between the mesh quality, computational accuracy, and resource consumption, the mesh with approximately 1.7 million elements was selected for the subsequent simulations.

### 2.5. Experimental Method

**Figure 4:**
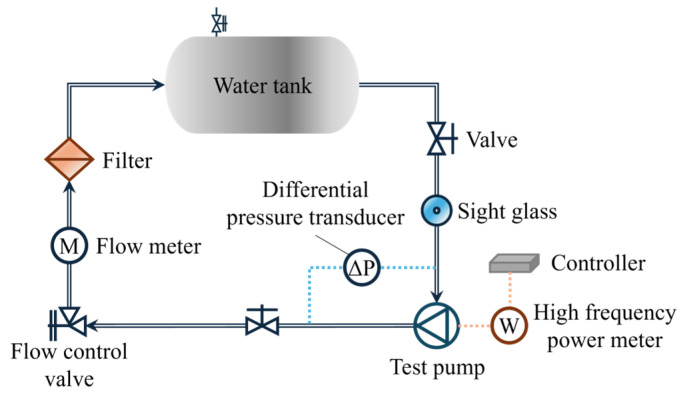
Closed-loop pump performance testing system.

**Figure 5:**
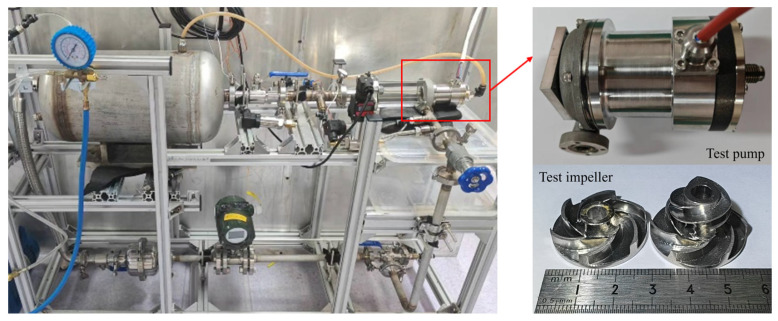
Test system and test pump.

A closed-loop pump performance testing system was established, in which water circulates within the system driven by the test pump. The schematic of the experimental setup is depicted in Figure 4. The rotational speed was controlled and monitored using a potentiometer in combination with a variable frequency drive. The flow rate was regulated by an angle valve at the pump outlet and measured using a turbine flow meter. A transparent pipe was connected to the water tank to monitor the liquid level. An air pressure tank, safety valve, and vacuum pump were installed above the water tank to adjust and stabilize the inlet pressure. Pressure transducers, differential pressure transducers, and temperature sensors were placed at key locations to acquire real-time data on the pressure and temperature within the system. Table 1 provides comprehensive information on the sensors used in this study. The uncertainty in the head measurement was calculated to be 0.707%. Photographs of the experimental setup and the MHCP are shown in Figure 5.

## 3. Analysis of Computational and Experimental Results

### 3.1. Validation of Numerical Model

**Figure 6:**
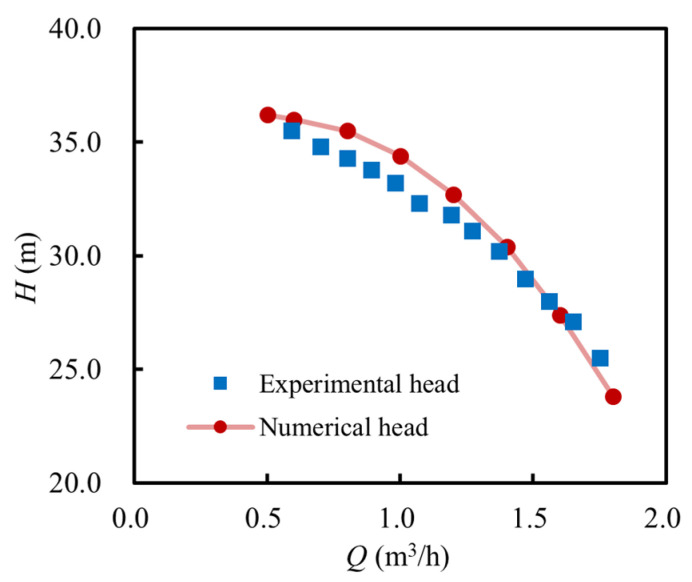
Hydraulic performance of the MHCP with an inducer.

Figure 6 illustrates the hydraulic performance curve of the MHCP (with an inducer) at the design speed (19,000 rpm) under different flow rates, obtained from both numerical simulations and experimental tests. The simulation results closely match the experimental data regarding the overall trends. Minor discrepancies in the head values are observed, primarily due to the uncertainties arising from prototype manufacturing and assembly tolerances. Nevertheless, these deviations remain within an acceptable range. The numerical model is considered reliable, as the largest head discrepancy compared to the experimental data is 1.6 m, yielding an error below 5%.

**Figure 7:**
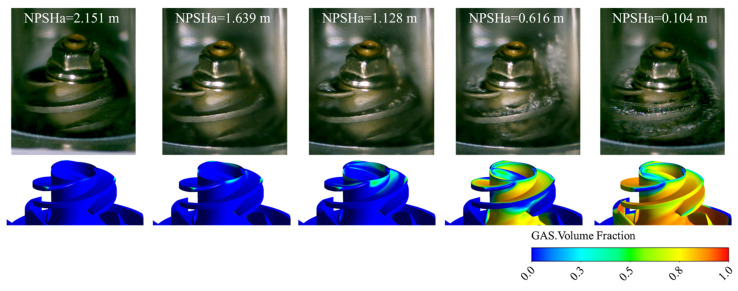
Validation of numerical model accuracy (comparison with visualization experiments by Zhou et al. [23]).

To further demonstrate the feasibility of the numerical model, Figure 7 presents the vapor volume fraction distributions within the pump under different NPSHa conditions. These results are compared with the cavitation flow patterns obtained from the visualization experiments conducted by Zhou et al. [23]. The simulation results align well with the experimental observations, confirming the reliability of the numerical model for representing the cavitating flow.

### 3.2. Effect of the Inducer on Flow Behavior in the MHCP

**Figure 8:**
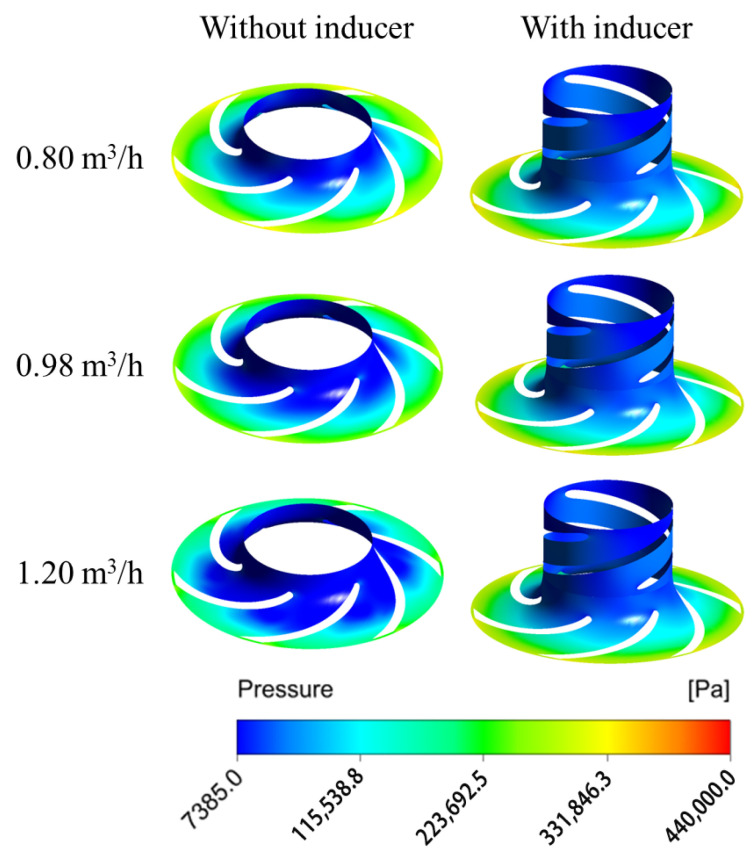
Pressure distribution inside the MHCP at span = 0.5.

**Figure 9:**
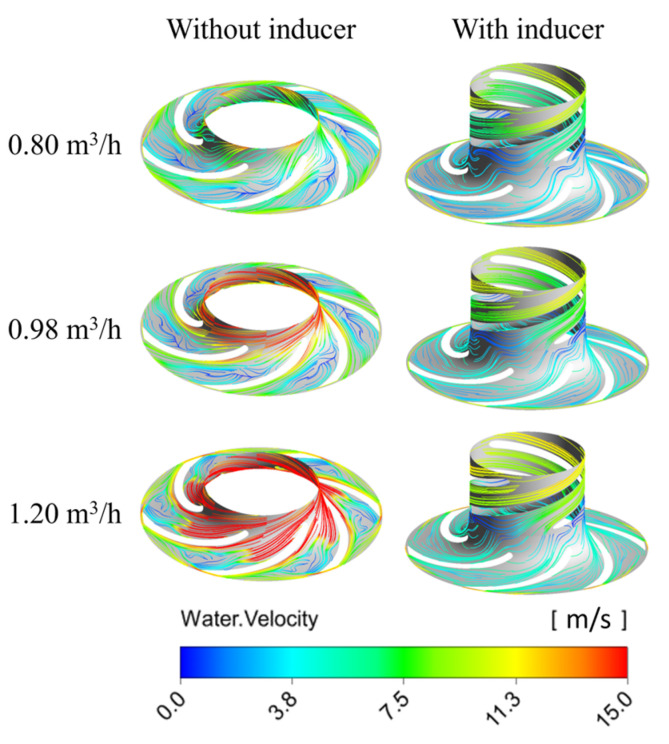
Streamline distribution inside the MHCP at span = 0.5.

Figure 8 and Figure 9 illustrate the pressure and streamline distributions at the mid-section of the rotating domain under various flow rates when the available net positive suction head (NPSHa) is 1.128 m. The results show that the pressure distribution within the impeller is similar across different flow rates: at a given impeller radius, the pressure on the non-working surface of the blade is consistently lower than that on the working surface, indicating a higher tendency for cavitation initiation on the non-working side. This pressure pattern also appears in the inducer, suggesting that cavitation originates on its non-working surfaces as well. Compared to the pump without an inducer, the inducer-equipped pump exhibits a higher overall pressure in the impeller region, demonstrating that the inducer effectively increases the inlet pressure and improves the cavitation resistance of the main impeller by enhancing its pressurization capability.

Additionally, a high-velocity zone is observed at the inlet of the pump without an inducer, which results from a drop in the static pressure and a rise in the dynamic pressure—conditions conducive to cavitation. A significant velocity drop and noticeable streamline distortion occur in the mid-passage of the impeller. Comparing the streamline patterns of the two configurations reveals that the inducer provides pre-pressurization to the incoming fluid, which reduces the inlet flow angle. As a result, the blade inlet angle of the impeller in the pump with an inducer can be appropriately reduced, contributing to smoother flow transitions and improved hydraulic performance.

### 3.3. Effect of the Inducer on the External Performance of the MHCP

**Figure 10:**
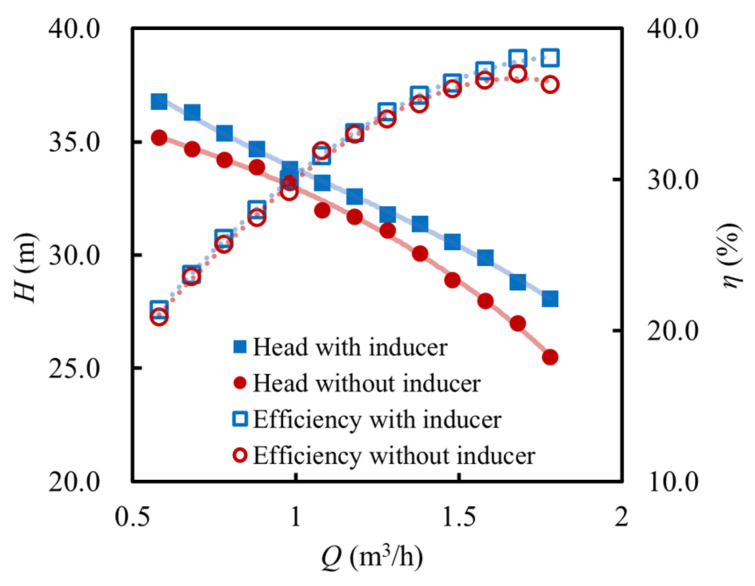
The influence of inducer on external performance of MHCP.

Figure 10 compares the experimentally obtained external performance curves of the MHCP with and without an inducer at the rated rotational speed. In this comparison, the head *H* is defined as the pressure difference between the pump outlet and inlet, while the efficiency *η* refers to the experimentally measured overall pump efficiency, including the motor, and is expressed as follows:

(5) $$H=\frac{\left(p_{out}-p_{in}\right)}{\rho g}$$

(6) $$\eta =\frac{\left(p_{out}-p_{in}\right)Q}{P_{motor}}$$

With an increase in the flow rate, the pump head gradually decreases, whereas the efficiency rises initially and then falls for both configurations. Although the presence of an inducer does not alter the general trend of the performance curves, it significantly enhances the hydraulic performance within the investigated operating range. Specifically, under the design conditions, the head of the MHCP with an inducer is 0.8 m higher than that without an inducer, and the maximum efficiency improvement reaches 1.5%. In the case without an inducer, cavitation tends to occur more readily at high flow rates, leading to a more rapid decline in the head. This is primarily due to the recirculation-induced pressure drop in the non-bladed region at the impeller inlet, which also results in a shift of the best efficiency point.

### 3.4. Analysis of Cavitation Characteristics

**Figure 11:**
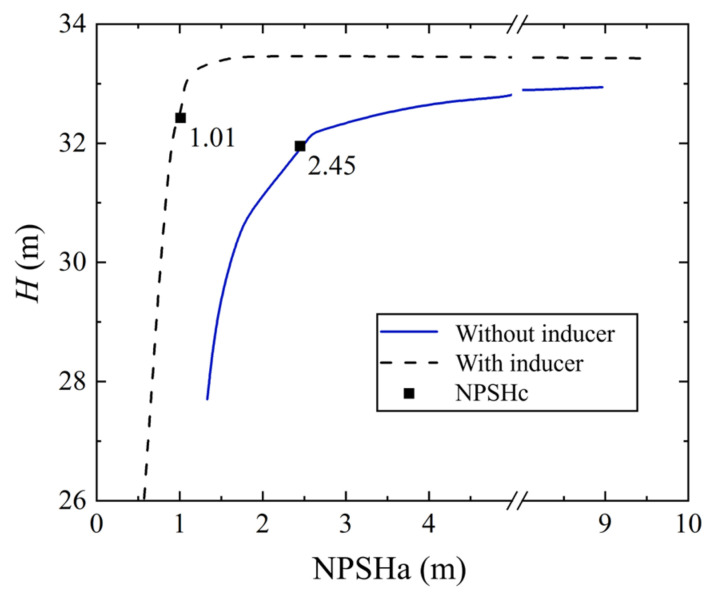
Cavitation performance curve of MHCP.

The experimentally measured cavitation performance curves at the rated operating conditions are presented in Figure 11. It can be observed that the MHCP head remains nearly constant before the onset of cavitation, after which it begins to decline. Assuming a 3% head drop as the criterion for defining the critical net positive suction head (NPSHc), the pump with an inducer exhibits a lower NPSHc, indicating improved cavitation resistance. However, once the critical cavitation point is reached, the head declines more rapidly in the pump with an inducer.

**Figure 12:**
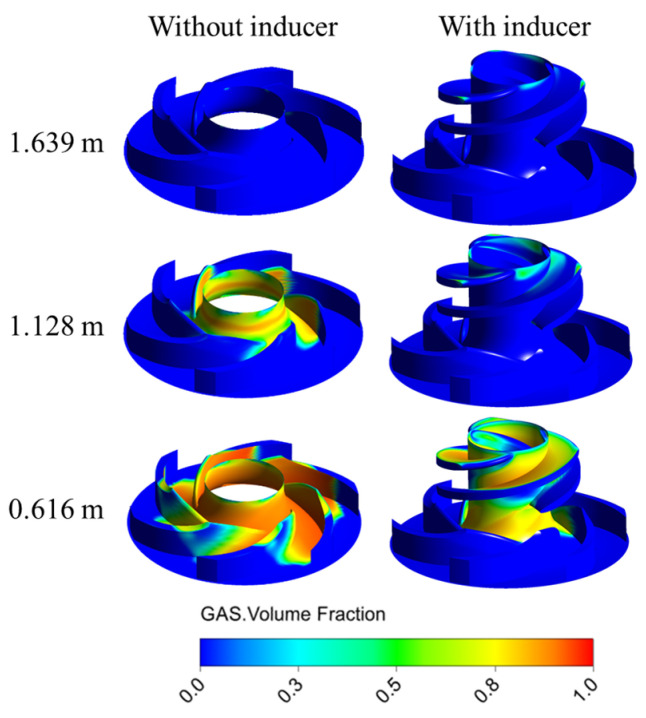
Influence of inducer on volume fraction distribution of MHCP under different NPSH conditions.

Figure 12 illustrates the vapor volume fraction distribution within the MHCP under various NPSHa conditions. For the pump without an inducer, cavitation initially occurs in the non-blade region at the impeller inlet. In contrast, for the pump with an inducer, cavitation first appears at the leading edge of the inducer blades. A detailed inspection of the impeller without an inducer reveals the presence of low vapor volume fraction zones near the leading edge, attributable to the formation of vortex cavities. These cavities arise from the tip leakage flow re-entering the inlet region and impinging on the incoming main flow, thereby generating local low-pressure zones. The comparative analysis clearly indicates that the presence of an inducer substantially improves the impeller’s resistance to cavitation by pre-pressurizing the inlet flow and mitigating the vortex-induced low-pressure effects in the non-bladed zone.

Under low NPSHa conditions, cavities rapidly grow and propagate downstream, leading to a swift blockage of the flow passages. The inducer, being an axial-flow impeller, possesses limited pressurization capability. Moreover, its blade inlet angle is relatively small, which intensifies the inlet backflow. Additionally, the tip leakage vortices from the inducer blades further promote cavitation development, resulting in stronger vortex structures. In the case of the MHCP, due to the relatively small impeller size, the bladeless region between the inducer and the impeller accounts for a larger proportion of the flow path. As a result, the pressure drop at the inducer trailing edge is more significant, which accelerates cavitation deterioration in the MHCP impeller. These characteristics are associated with increased turbulence intensity and flow instability, ultimately contributing to the abrupt drop in the head. This suggests that the inducer effectively enhances cavitation suppression. It also broadens the stable operating range before cavitation inception. However, after fully developed cavitation begins, the inducer may cause a more severe pressure drop near its trailing edge. This pressure drop can exacerbate cavitation deterioration.

## 4. Conclusions

This study investigates the internal characteristics, external performance, and cavitation characteristics of an MHCP with an inducer through a combination of numerical simulations and experimental measurements. The key conclusions can be summarized as follows:

(1) The numerical model demonstrates good predictive capability for MHCP performance when validated against experimentally obtained hydraulic performance data and flow visualization images. Within the investigated operating range, the addition of an inducer significantly enhances the external performance of the MHCP.
(2) The inducer provides pre-pressurization to the incoming fluid. The relatively high operating speed results in a higher circumferential velocity at the inducer inlet and a reduced inlet flow angle, which allows the main impeller to adopt a smaller blade inlet angle.
(3) The inducer alleviates the local low-pressure zones caused by the vortex cavities generated by the interaction between the tip leakage backflow and the main flow, thereby suppressing cavitation in the non-bladed region.
(4) Once the pump reaches the critical NPSH, the presence of an inducer leads to a more rapid degradation caused by cavitation. This is attributed to the relatively enlarged bladeless region downstream of the inducer’s trailing edge in the MHCP, which intensifies the local pressure drop and accelerates cavitation deterioration in the impeller.

## Figures and Tables

**Table 1:** Comprehensive information on the sensors.

Type	Range	Measurement Accuracy
PT100 (MICROSENSOR, Baoji, China)	−50 °C~250 °C	±0.5 °C
Differential pressure transducer (MICROSENSOR, Baoji, China)	0~1 MPa	±0.5%
Pressure transducer (MICROSENSOR, Baoji, China)	0~0.6 MPa	±0.5%
Turbine flowmeter (FIMEET, Hefei, China)	0.6~6 m^3^/h	±0.5%
Power meter (QINGZHI, Qingdao, China)	0~5 kW	±0.5%

## Data Availability

The original contributions presented in this study are included in the article. Further inquiries can be directed to the corresponding authors.

## Abbreviations

The following abbreviations are used in this manuscript:

MHCP	Miniature High-Speed Centrifugal Pump
NPSHr	Required Net Positive Suction Head
UAV	Unmanned Aerial Vehicle
NPSHa	Available Net Positive Suction Head
NPSHc	Net Positive Suction Head
